# Carbide Derived Carbon (CDC) as novel adsorbent for ibuprofen removal from synthetic water and treated sewage effluent

**DOI:** 10.1007/s40201-020-00554-0

**Published:** 2020-10-09

**Authors:** Ismail W. Almanassra, Viktor Kochkodan, Guhankumar Ponnusamy, Gordon Mckay, Muataz Ali Atieh, Tareq Al-Ansari

**Affiliations:** 1 Division of Sustainable Development, College of Science and Engineering, Hamad Bin Khalifa University, Qatar Foundation, Doha, Qatar; 2 Qatar Environment and Energy Research Institute, Hamad Bin Khalifa University, Qatar Foundation, PO Box 5825, Doha, Qatar; 3 Doha, Qatar; 4 College of Engineering, University of Sharjah, PO Box 27272, Sharjah, United Arab Emirates; 5 Desalination Research Group, Research Institute of Sciences and Engineering, University of Sharjah, PO Box 27272, Sharjah, United Arab Emirates; 6 Division of Engineering Management and Decision Sciences, College of Science and Engineering, Hamad Bin Khalifa University, Qatar Foundation, Doha, Qatar

**Keywords:** Adsorption, Ibuprofen, Carbide derived carbon, Treated sewage effluent

## Abstract

**Purpose:**

Pharmaceuticals are becoming one of the largest environmental concerns when it comes to the water treatment industry. Increased usage of these chemicals poses a serious risk to ecology and human health due to their leakage into surface waters. In the present study, carbide derived carbon (CDC) was used for the first time as a new adsorbent to remove ibuprofen from synthetic water and wastewater effluent.

**Methods:**

The morphology, chemical composition, surface area and surface charge of the CDC particles were investigated using the transmission electron microscopy, scanning electron microscopy, energy dispersive spectroscopy, Fourier transform infrared spectroscopy, BET analysis and zeta potential measurements. The effects of CDC dosage, temperature, initial pH and agitation speed on the adsorption process were examined by using batch adsorption experiments. Moreover, the adsorption kinetics, thermodynamics, and isotherms were investigated.

**Results:**

Adsorption and kinetic equilibrium data demonstrate that the adsorption of ibuprofen onto the CDC obeys the Langmuir isotherm model and the kinetics follow the pseudo-2nd order mechanism. The thermodynamic results reveal that ibuprofen adsorption is endothermic and spontaneous. The ibuprofen removal by CDC was mainly controlled by the electrostatic forces at high pH of the feed solution and by the dispersive interactions in acidic media. The ibuprofen removal is promoted at high temperature, high agitation speed and low pH. The highest adsorption capacity of ibuprofen onto the CDC was 367 mg/g at pH 3. Furthermore, the CDC efficiently removed ibuprofen from spiked treated sewage effluent.

**Conclusions:**

The obtained data indicate that the CDC provides a fast and efficient adsorptive removal of ibuprofen both from a model aqueous solution and treated sewage effluent.

## Introduction

There is a rising societal awareness and concern about the increased environmental pollution in water bodies [1]. The emerging contaminants in such delicate ecosystems are increasing in diversity in terms of type and source where the origins can be from insecticides, herbicides, domestic wastes, food processing waste, volatile organic compounds, chemical waste and pollutants from livestock operations [2]. There is added emphasis on pollutants from pharmaceutical and personal care products (PPCP) due the inability of traditional waste-water purification plants to completely remove these pollutants from water [3, 4]. The consumption of PPCP’s is increasing as they are primarily found in a variety of different applications such as aquaculture, farming, livestock medicine and in everyday human life [5]. Usually chemicals from these products are found in water in trace concentrations (ng/L or µg/L). However, there is a concern as information related to the term exposure to these chemicals is limited [6, 7]. Currently, research to address such challenges is related to defining the maximum permitted levels and the control methods for these pollutants in water [8–10].

Ibuprofen is one of the most broadly used pharmaceuticals worldwide and listed as a set medicine for its antipyretic effects, anti-inflammatory analgesic, and non-steroidal used for fever, pain and rheumatic disorders treatment. It has a slight solubility in aqueous solutions and has good mobility in the marine environment [5, 11, 12]. For instance, Ibuprofen one of the top five used medicines in the United Kingdom and found in detectable limits in sewage and wastewater treated effluents [13]. According to European and US regulating authorities, the available medicines are subjected to poisoning tests when the level of the active pharmaceutical constituent exceeds 10 ng/L (European Medicines Agency) or 1 mg/L (US legislation) in water [14]. It has been reported by different countries that ibuprofen has been detected in wastewater treated sewage effluents in concentrations of 60 ng/L to 100 µg/L [13, 15–18].

The treatment of waters containing ibuprofen was investigated by utilizing different technologies such as; ozonation, activated sludge systems and adsorption, the studied variables and the performance of ibuprofen removal by these methods have been discussed previously [19]. Amongst the potential techniques for wastewater treatments, the adsorption process is considered one of the best efficient methods for the wastewater purification that contains organic contaminants and pharmaceutical pollutants. The adsorption process has some advantages amongst other methods since it requires a relatively simple design, low investment costs and provides high removal efficiency [20].

The removal of ibuprofen from aqueous solutions has been investigated using several types of adsorbents including carbon nanotubes (CNTs) [13, 21], graphene oxide nanoplatelets [22], activated carbon (AC) [8, 12, 15, 23–26] and nano clay composite [27]. The removal of ibuprofen by carbon based materials depends on the preparation method and the surface area of the adsorbent. For example; Guedidi et al. [24] found that the thermally treated AC was found to be promoting the ibuprofen removal at low pH. Mestre et al. [25] reported that ACs prepared by chemical activation using K_2_CO_3_ and steam activation have more advantages in terms of adsorption capacity and adsorption rate than AC, synthesized using only the chemical activation, due to the formation of a super microporous configuration by a two-step method rather than one. Most researchers agree that chemical oxidation of carbon based adsorbents has a negative impact on the removal of ibuprofen from aqueous solutions because of decreasing micro-pore volume or the blockage of pore openings by the formed functional groups [8, 13, 24]. The ACs showed a limited adsorption capacity of ibuprofen due to the negative functional groups found on its surface in nature, for instance; Delgado et al. [26] obtained an adsorption capacity of 70 mg/g using F400 and Picabiol ACs. Banerjee et al. [22] found that graphene oxide nano-pellets have an adsorption capacity of 6 mg/g at pH 6. Although some materials showed a higher adsorption capacity of 231.5 mg/g for single-wall CNT [13], the search of new types of adsorbents is still under investigation.

Carbide derived carbon (CDC) is an amorphous to crystalline carbon material produced by thermal treatment of different kind of carbides such as TiC and SiC [28–30]. The surface properties of the CDC such as porosity, surface area, shape and pore size distribution can be tuned by varying the preparation temperature and the feedstock used for production [31–33]. The highest reported surface area of CDC is 3116 m^2^/g [34], which is higher than the surface area of AC and CNT [35, 36]. Due to the CDC structural and tunable properties, it can provide more binding sites that might improve the adsorption capacity of different pollutants [37]. In this regard, CDCs were utilized for the adsorptive removal of cytokines [38], acetaldehyde [39], carbon dioxide [40], different electrolytes [41] and phosphate [42]. Moreover, Alvarez et al. [43], in their turn explored the kinetics and isotherms of the removal of diclofenac and metronidazole from water solutions using CDC produced from TiC powder at various synthesis temperatures (800–1400 °C). The highest surface area of the CDC found at the synthetic temperature 800 and 1000 °C. The CDCs demonstrated fast kinetics and extremely high adsorption capacities of diclofenac and metronidazole. Bearing in mind the previous findings, the CDC could be a promising and efficient adsorbent for the removal of a variety of pollutants. To the best of our knowledge, the potential removal of ibuprofen from aqueous solutions using CDC, and the influence of adsorption parameters such as initial pH, temperature, adsorbent dose and agitation speed on the ibuprofen adsorption by CDC have not been reported.

The objective of this study is to examine the ibuprofen adsorption from synthetic water and treated sewage effluent (TSE) by a commercial CDC synthesized from TiC powder at 800 °C. The CDC was fully characterized and investigated for ibuprofen uptake by varying the adsorption parameters namely; temperature, initial pH, agitation speed, initial ibuprofen concentration and adsorbent dosage. Furthermore, the thermodynamics, isotherms and kinetics of the ibuprofen adsorption via CDC were studied, and finally, the potential application of CDC for ibuprofen removal from spiked local TSE was investigated.

## Materials and methods

### CDC characterization and synthesis

A commercial CDC was used in the current study was purchased from the Carbon-Ukraine Company (Y-Carbon ltd (Type: Nanopore U). The synthesis process of the CDC as described by the manufacturer is as follows [44]; briefly; the TiC powder was initially heated to 800 °C under a flow of argon, then it was treated with chlorine and hydrogen gases as follows; Cl_2_ 800 °C for 6.5 h following by H_2_ 600 °C for 2 h. The chemical composition and surface morphology of the CDC was studied through field emission scanning electron microscope (FE-SEM, QUANTA FEG 650, Thermo Fisher Scientific, USA) operated with energy dispersive spectroscopy (EDS, Bruker Xflash 6l60, Germany) used for elemental analysis. Transmission electron microscope (TEM) pictures were acquired using (FEI Talos F200X TEM microscope) functioning at 200 kV. For TEM imaging, a sample of CDC powder was immersed in isopropyl alcohol by ultra-sonication for 5 min, then a drop of 20 µl of the spread solution was placed on 300 mesh lacey copper carbon grid then it has been dried at room temperature before imaging. The chemical groups on the CDC surface was evaluated by Fourier transform infrared spectroscopy (FTIR) (Thermo Fisher Scientific Nicolet iS10), the spectrum of the FTIR was between 600 and 4000 cm^− 1^. The BET surface area analyzer Micromeritics (ASAP 2020, Norcross, USA) operating with N_2_ gas adsorption at a temperature 77K was used for surface area measurements and pore size distribution. Zetasizer type (Nano ZS 90, Malvern Instruments Ltd., UK) functioning with a 4.0 MW internal laser was utilized for the zeta potential readings of CDC particles, the measurements were recorded at temperature 25 °C by varying the solution pH between 3 and 9. Zeta potential values have been measured three times at each pH and average values are presented.

### Ibuprofen preparation

**Fig. 1:**
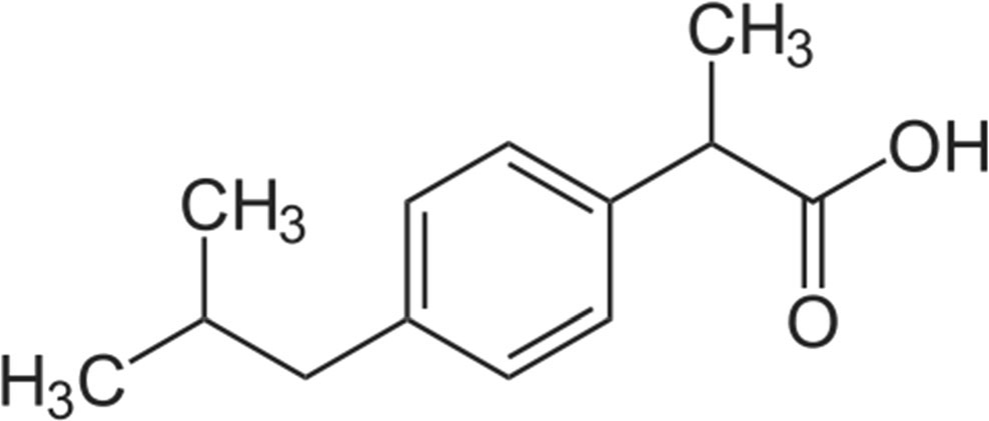
Ibuprofen molecular structure

The ibuprofen powder employed in the current study was acquired from Sigma Aldrich (China), the molecular structure is illustrated in Fig. 1, the physiochemical properties of ibuprofen are as follows; molecular weight 206.28 g/mol, purity ≥ 98%, acid dissociation constant; pKa 4.91 [45], moderately soluble in water (Maximum solubility 21 mg/L) and highly soluble in most of organic solvents [46, 47]. In this study, a stock of 20 ppm of ibuprofen aqueous mixture was produced by immersing the required mass of ibuprofen in ultra-high quality distilled water (18.2 MΩ). Then the mixture was sonicated under ice cooling for 1 h using 750 Watt, 20 kHz Cole Parmer sonicator at a pulse 3:1 and 40% amplitude. For the adsorption experiments, the ibuprofen mixtures of the required concentrations were produced from the 20 ppm stock mixture by suitable dilution. The pH values of the ibuprofen solutions were monitored by the addition of 0.1M NaOH or HCl purchased from Sigma Aldrich® (India) and Honeywell Fluka® (Austria), respectively.

For the TSE adsorption experiments, a 1 ppm of ibuprofen was spiked in the TSE under similar sonication conditions. The local TSE used in this study was obtained from the Doha North wastewater purification facility in north Doha, Qatar.

### Adsorption experiments

The adsorption tests were performed by means of batch method adsorption tests. In all experiments, the required quantity of CDC was added to a volume of 100 ml of the ibuprofen mixture in Erlenmeyer flasks, agitated at the required conditions and samples for analysis were collected with time. VMR standard analog shaker (Model 5000, USA) was used for adsorption experiments at 20 °C and Grant OLS Aqua Pro temperature controlled shaker bath (Model OLS26, UK) was used for the high temperature experiments. The effects of adsorption parameters were investigated by varying the CDC loading from 5 to 400 mg, ibuprofen feed concentration (1–20 mg/L), pH (3–9), temperature (293–318 K), agitation speed of 100–200 RPM and adsorption time of 1 min – 24 h. The pH of the ibuprofen solutions was controlled through the addition of HCl (0.10 M) or NaOH (0.10 M). During the adsorption, each taken sample was filtered using 0.45 micron membrane filtration and the ibuprofen content was examined by ultra-high performance liquid chromatography (UHPLC-DAD), (Agilent Technologies, Infinity series LC, 1260, USA) equipped with Acclaim 120, C18, 3 µm Analytical (4.6 × 150 mm) Column (Thermo scientific, USA). The ibuprofen analytical standard was used for UHPLC-DAD calibration and quantification. The UHPLC operating conditions are provided in Table 1. The limit of quantification (LOQ) and the limit of detection (LOD) for ibuprofen was found to be 20.6 µg/L and 6.2 µg/L using the empirical method. From the concentrations obtained through the analysis, values below LOD were considered as zero. For the values between LOQ and LOD, concentrations were approximated based on the peaks obtained in comparison to the LOQ peak. To ascertain the repeatability and reproducibility of the results, each adsorption experiment was performed three times and the average values were presented in the figures. The maximum error observed was ± 5%.

**Table 1:** UHPLC operating conditions

Parameters	Conditions
Solvent A	Milli-Q water with 10 mM H_3_PO_4_ (> 85%)
Solvent B	Methanol (≥ 99.9%) HPLC grade
Injection volume (µL)	100
Run time (minutes)	8
Flowrate (mL/min)	0.8
Temperature (°C)	35
Wavelength (nm)	224
Isocratic elution	15% A and 85% B

The adsorption values (*q*_*e*_, mg of ibuprofen/g of CDC) of ibuprofen uptake by CDC and the ibuprofen percentage removal (RE) from water was estimated from the given equations;

1 $${q}_{e}=\frac{\left({C}_{i}-{C}_{e}\right)\times V}{W}$$

2 $$RE\left(\%\right)=\frac{\left({C}_{i}-{C}_{e}\right)}{{C}_{i}}\times 100$$

where, *C*_*i*_ is the initial ibuprofen concentration (mg/L), *C*_*e*_ is the last concentration of the residual ibuprofen in the experiment, *V* is the volume of the ibuprofen solution (L) and *W* is the mass of the CDC (g).

### Adsorption kinetics

The kinetics and sorption equilibrium studies produce important parameters in process design for real applications. These values can predict and provide an insight on the rate limiting step of the pollutant rate of adsorption and the maximum adsorption uptake [48]. The experimental adsorption results were fitted by four adsorption kinetic models; Lagergren pseudo-1st -order, the pseudo-2nd -order, the Elovich model and the intra-particle diffusion model. The mathematical expressions, linearized forms and plots of the mentioned models are tabulated in Table 2.

**Table 2:** List of kinetic adsorption models

Kinetic model	Model formula	Linearized formula	XY Scheme	Ref
Pseudo-1st -order	$\frac{{dq}_{t}}{dt}={k}_{1}\left({q}_{e}-{q}_{t}\right)$	$log\left({q}_{e}-{q}_{t}\right)=log\left({q}_{e}\right)-\left(\frac{{k}_{1}}{2.303}\right)t$	$log\left({q}_{e}-{q}_{t}\right)$ vs $t$	[49]
Pseudo-2nd -order	$\frac{{dq}_{t}}{dt}={k}_{2}{\left({q}_{e}-{q}_{t}\right)}^{2}$	$\frac{t}{{q}_{t}}=\frac{1}{{k}_{2}{q}_{e}^{2}}+\left(\frac{1}{{q}_{e}}\right)t$	$\frac{t}{{q}_{t}}$ vs $t$	[50]
Elovich model	$\frac{{dq}_{t}}{dt}=\alpha exp\left(-\beta {q}_{t}\right)$	${q}_{t}=\frac{1}{\beta }ln\left(\alpha \beta \right)+\left(\frac{1}{\beta }\right)ln\left(t\right)$	${q}_{t}$ vs $ln\left(t\right)$	[51]
Intra-particle diffusion	${q}_{t}={k}_{diff}\sqrt{t}+c$	${q}_{t}={k}_{diff}\sqrt{t}+c$	${q}_{t}$ vs $\sqrt{t}$	[52]

Where, *q*_*t*_ is the quantity of ibuprofen removed by the CDC at any time (mg/g), t is the time (min), *q*_*e*_ is the quantity of ibuprofen adsorbed onto the CDC at equilibrium (mg/g), *k*_*1*_ is the pseudo 1st order rate constant (min^− 1^), *k*_*2*_ is the pseudo 2nd order rate constant (g/mg/min), *β* is the constant of desorption (g/mg), *α* is the initial rate of adsorption (mg/g/min), *k*_*diff*_ is the intra-particle diffusion rate parameter (mg/g/min^− 0.5^), *c* is the intraparticle intercept (mg/g).

### Adsorption isotherms

Several isotherm models such as Langmuir, Temkin, Freundlich and Dubinin-Radushkevich (DR) were used to fit and study the adsorption data. The isotherm models and their linearized forms are listed in Table 3.

**Table 3:** Adsorption isotherms

Isotherm	Model	Linearized form	XY Plot	Ref
Langmuir	${q}_{e}=\frac{{X}_{m}h{C}_{e}}{\left(1+h{C}_{e}\right)}$	$\frac{{C}_{e}}{{q}_{e}}=\frac{1}{{X}_{m}h}+\frac{{C}_{e}}{{X}_{m}}$	$\frac{{C}_{e}}{{q}_{e}}$ vs ${C}_{e}$	[53]
Temkin	${q}_{e}=\frac{RTln\left({A}_{t}{C}_{e}\right)}{b}$	${q}_{e}=\frac{RT}{b}ln{A}_{t}+\frac{RT}{b}ln{C}_{e}$ Or ${q}_{e}=Bln{A}_{t}+Bln{C}_{e}$	${q}_{e}$ vs $ln{C}_{e}$	[54]
Freundlich	${q}_{e}={K}_{F}{C}_{e}^{\frac{1}{n}}$	$ln{q}_{e}=ln{K}_{F}+\frac{1}{n}ln{C}_{e}$	$ln{q}_{e}$ vs $ln{C}_{e}$	[55]
Dubinin Radushkevich	${q}_{e}=\left({q}_{s}\right)exp\left(-{k}_{ad}{\varepsilon }^{2}\right)$	$ln\left({q}_{e}\right)=ln\left({q}_{s}\right)-{k}_{ad}{\varepsilon }^{2}$	$ln\left({q}_{e}\right)$ vs ${\varepsilon }^{2}$	[56]

Where, *h* is the equilibrium or Langmuir constant and provides an indication of the extent of ibuprofen adsorption affinity, *X*_*m*_ is the maximum ibuprofen adsorption uptake (mg/g). *R* is the ideal gas constant (J/mole/K) in Temkin model, *T* is the temperature (K), *A*_*t*_ is the adsorption capacity coefficient or the constant of equilibrium binding (L/mg), *b* is the energy of adsorption coefficient or Temkin constant, the term *B = RT/b*, is a constant belongs to the heat of adsorption. *K*_*F*_ is the adsorption capacity coefficient (mg/g/(mg/L)^1/n^ and *n* is the adsorption intensity coefficient, are Freundlich constants. *q*_*s*_ is the theoretical highest uptake (mg/g) in the Dubinin Radushkevich (DR) isotherm, *k*_*ad*_ is activity coefficient or DR constant term (mol^2^/J^2^) and *ε* is the Polayni potential (J/mol) determined through the given formula;

3 $$\varepsilon =RTln\left(1+\frac{1}{{C}_{e}}\right)$$

The Langmuir model assumes; (1) the adsorption occurs at a specific identical homogeneous site on the adsorbent surface, (2) uniform and constant energies for monolayer adsorption. Once adsorption is completed on the surface of the adsorbent, adsorption no longer occurs on occupied sites. An important dimensionless parameter obtained from Langmuir model called separation factor R_L_ representing the adsorption favorability, if *R*_*L*_>1 the adsorption is unfavorable, *R*_*L*_=0 irreversible, *R*_*L*_=1 linear, and for favorable adsorption *R*_*L*_ is in the range 0 to 1 [57]. *R*_*L*_ is given in the following equation, where *C*_*0*_ is the maximum initial loading of the adsorbate in (mg/L).

4 $${R}_{L}=\frac{1}{1+h{C}_{0}}$$

The Temkin isotherm takes into account that the energy of adsorption reduces linearly throughout the adsorption process rather than reduces exponentially. The Freundlich isotherm represents the reversible and non-ideal adsorption and it is not only related to the monolayer formation. The Freundlich model adopts that the adsorbent surface is heterogeneous where no consideration of saturation capacity and the adsorbed amount increased with increasing the feed concentration of the adsorbate. The DR model is applied to reveal the adsorption mechanism by calculating the mean free energy (*E*). If *E* is less than 8 kJ/mol the adsorption process is dominated by physisorption, while it is chemical ion exchange mechanism if *E* value between 8 and 20 kJ/mol [58]. *E* is calculated by applying the given formula;

5 $$E=\frac{1}{\sqrt{2{k}_{ad}}}$$

With the purpose of determining the best optimum model to describe the experimentally determined data, different statistical factors can be used. In our investigation, the determination coefficient (R^2^), chi-square test (χ^2^) and the sum of the square of the errors (SSE) were utilized to obtain the optimum model, given as follows:

6 $${{\upchi }}^{2}={\sum\limits }_{i=1}^{n}\frac{{\left({q}_{i\left(\mathrm{e}\mathrm{x}\mathrm{p}\right)}-{q}_{i\left(cal\right)}\right)}^{2}}{{q}_{i\left(\mathrm{e}\mathrm{x}\mathrm{p}\right)}}$$

7 $$SSE={\sum\limits }_{i=1}^{n}{\left({q}_{i\left(\mathrm{e}\mathrm{x}\mathrm{p}\right)}-{q}_{i\left(cal\right)}\right)}^{2}$$

where, q_i(exp)_ is the experimental adsorption uptake gained from the experimental tests (mg/g) and q_i(cal)_ is the adsorption uptake (mg/g) gained from the model.

### Adsorption thermodynamics

The removal of ibuprofen by adsorption using CDC was conducted by varying the temperatures to evaluate the thermodynamic characteristics i.e., the entropy ($\Delta {S}^{^\circ}$) change, enthalpy ($\Delta {H}^{^\circ }$) changes and Gibbs free energy change ($\Delta {G}^{^\circ }$). The Gibbs free energy indicates evidence regarding the feasibility and spontaneity of the adsorption process and it can be calculated at each temperature from the following formula;

8 $$\Delta {G}^{^\circ }=-RTln{K}_{d}$$

where; ${K}_{d}$is the adsorption equilibrium constant [12, 59] and calculated using;

9 $${K}_{d}=\frac{{C}_{a}}{{C}_{e}}$$

where; ${C}_{a}$ is the quantity of ibuprofen adsorbed on the CDC at the equilibrium condition (mg/L). The change in enthalpy provides the information regarding whether the adsorption process is endothermic or exothermic, while the entropy change indicates the extent of order/disorder or the degree of freedom in the system. Entropy and enthalpy changes are obtained by calculating the slope and the intercept of the following formula by sketching $ln{K}_{d}$ and$\left(\frac{1}{T}\right)$ [60];

10 $$ln{K}_{d}=\left(\frac{\Delta {S}^{^\circ }}{R}\right)-\left(\frac{\Delta {H}^{^\circ }}{R}\right)\left(\frac{1}{T}\right)$$

## Results and discussion

### Characterization of CDC

**Fig. 2:**
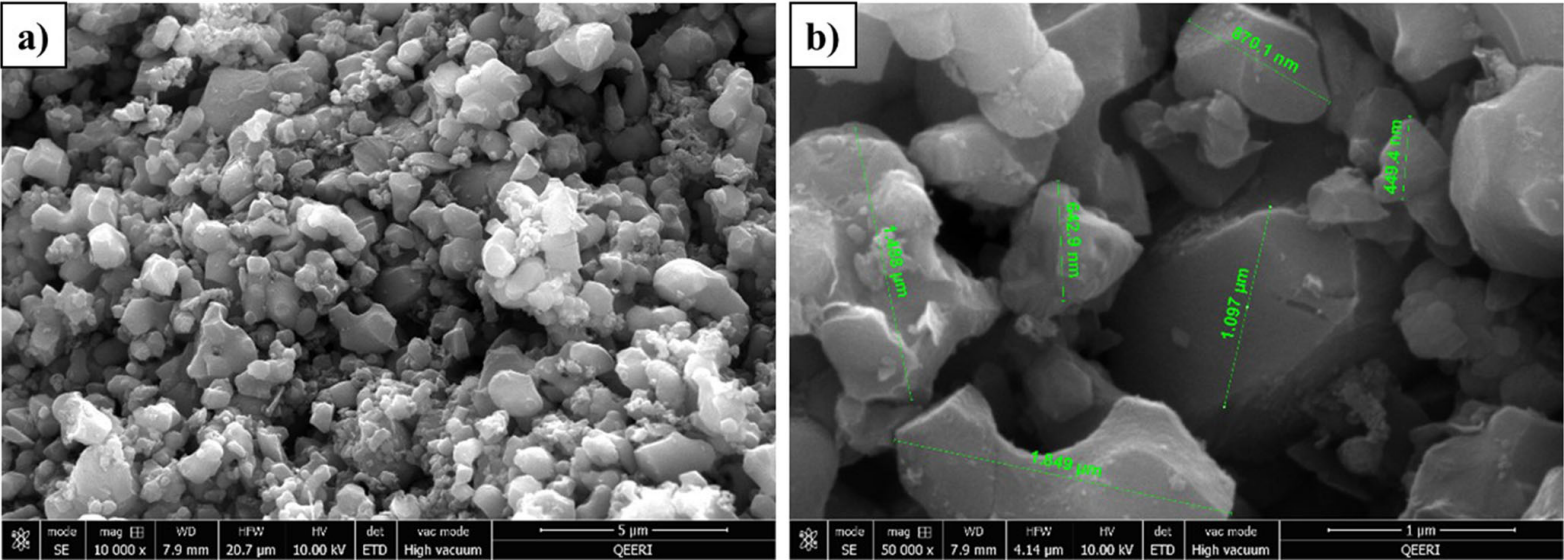
SEM images of CDC powder, (**a**) at low magnification (**b**) at high magnification

**Fig. 3:**
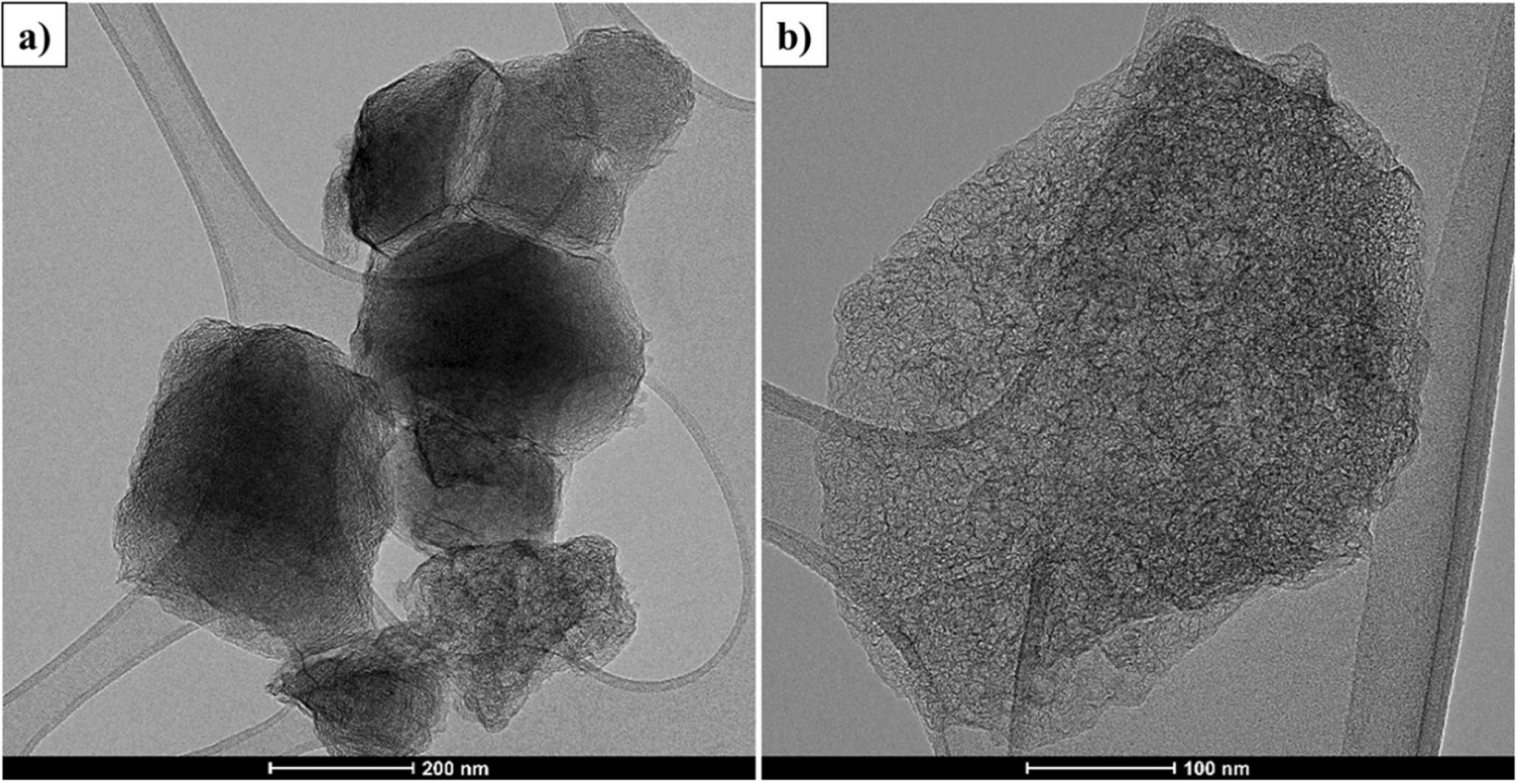
TEM images of CDC powder, (**a**) at low magnification (**b**) at high magnification

The surface morphology of the CDC adsorbent was investigated by SEM. Figure 2 displays the SEM images of CDC at high and low magnifications. It can be observed that, the CDC has homogeneous flat surfaces with irregular shaped particles having a wide range of sizes from 200 nm – 2 µm. In some areas the particles appear to be agglomerated with each other forming an irregular sphere similar to a graphitic shape. The elemental analysis by the EDS illustrates that the main constituents of the CDC are carbon and oxygen with trace amounts of the titanium and chlorine (91.86% C, 4.56% O, 2.67% Cl and 0.91% Ti). Figure 3 displays the TEM pictures of the CDC, where the developed CDC is a highly porous material as seen in Fig. 3b.

**Fig. 4:**
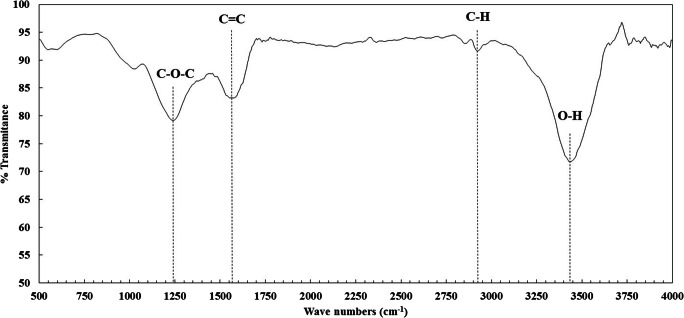
FTIR spectrum of CDC

**Fig. 5:**
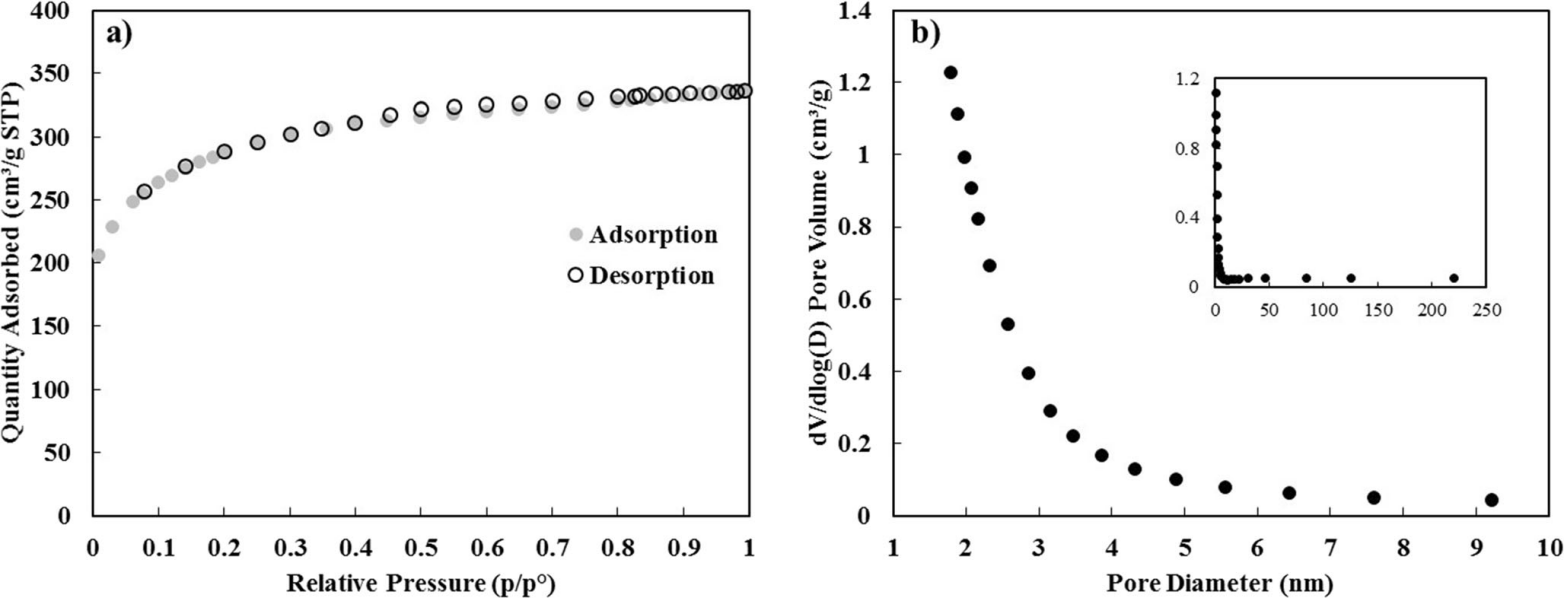
(**a**) N_2_ adsorption-desorption isotherm of CDC, (**b**) BJH pore size distribution curve

The FTIR examination was conducted to explore the functional groups and surface characteristics of the CDC. Figure 4 illustrates the FTIR spectrum of the CDC, with a broad water peak at 3430 corresponding to the O-H stretching [61], three more characteristic peaks were detected at 2922, 1574 and 1241 cm^− 1^ corresponding to C-H, C = C and C-O-C stretches, respectively [62]. The specific surface area of the adsorbent is related to the available adsorptive cites and was determined by the BET method. It has been reported that the surface area of the CDC synthesized from TiC powder varies with temperature reaching a maximum value at 800–1000 °C and then falls with increasing the synthetic temperature [28]. This is because of the graphitization of CDC at elevated temperature [63]. For the used CDC in the current study, the BET surface area was found to be 1054 m^2^/g. The N_2_ adsorption-desorption curve of CDC is depicted in Fig. 5a. The structure of the isotherm is alike to type *Ia* sorption isotherm according to IUPAC categorization [64]. Type *Ia* materials suggesting a highly microporous material, it also suggests that the amount of the adsorbed material is controlled by the surface of the adsorbent not by the internal surfaces of the pores. The distribution of pore sizes of the CDC was evaluated according to the Barrett, Joyner, and Halenda (BJH) method. Figure 5b demonstrates that most of the pores of the CDC particles are in the range of 1–4 nm with a high peak at 2 nm, the BJH adsorption and desorption results show that the average pore diameter of the CDC is 2.8–3.1 nm, confirming the adsorption is primarily on the adsorbent surface rather than on the internal pore surfaces.

### Adsorption data

**Fig. 6:**
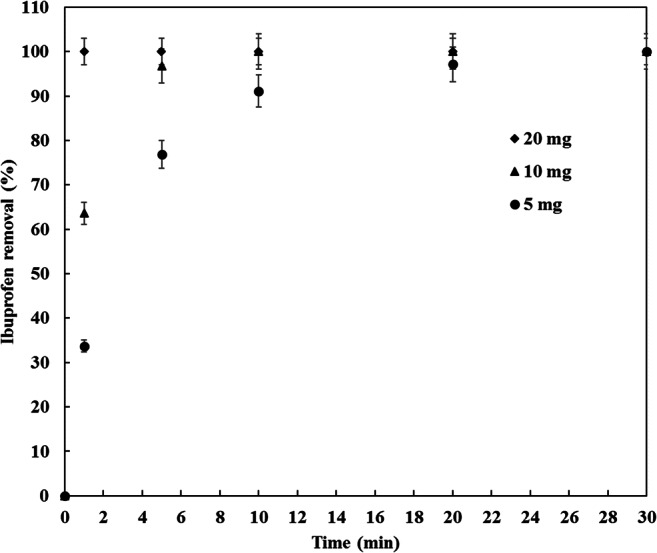
Ibuprofen removal at different CDC dosage, (agitation speed, 150 RPM; temperature, 20 °C, pH 5)

At the beginning of this study, CDC dosages ranging from 5 to 400 mg were added to 100 ml of 1 ppm ibuprofen solution. Figure 6 illustrates the ibuprofen removal with time at different CDC loadings. The ibuprofen removal was increasing with contact time. As depicted and regardless of the dosage of CDC, the rate of ibuprofen removal was initially very fast and increased slightly with time until complete removal was obtained. The rapid adsorption process attributed to the surface area of the CDC which offers a high number of available adsorption sites. These vacant active adsorption sites becoming occupied with time until saturation. Another reason of the fast adsorption process is attributed to the higher PZC of the CDC as demonstrated in the effect of pH section. The ibuprofen removal was found to be faster for higher dosages of CDC achieving a complete removal of ibuprofen after 1 min for a CDC dosage of 20 mg or above explained by the huge number of vacant sites. A complete removal of ibuprofen was also obtained for low dosage of CDC, 5 mg of CDC reached equilibrium in 30 min and 10 mg required 10 min for a the complete removal of 1 mg/L of ibuprofen. In term of material savings, the dose 5 mg in 100 ml of ibuprofen solution (i.e. 0.05 g/L) was selected for the rest of the experiments.

### Adsorption isotherms

**Fig. 7:**
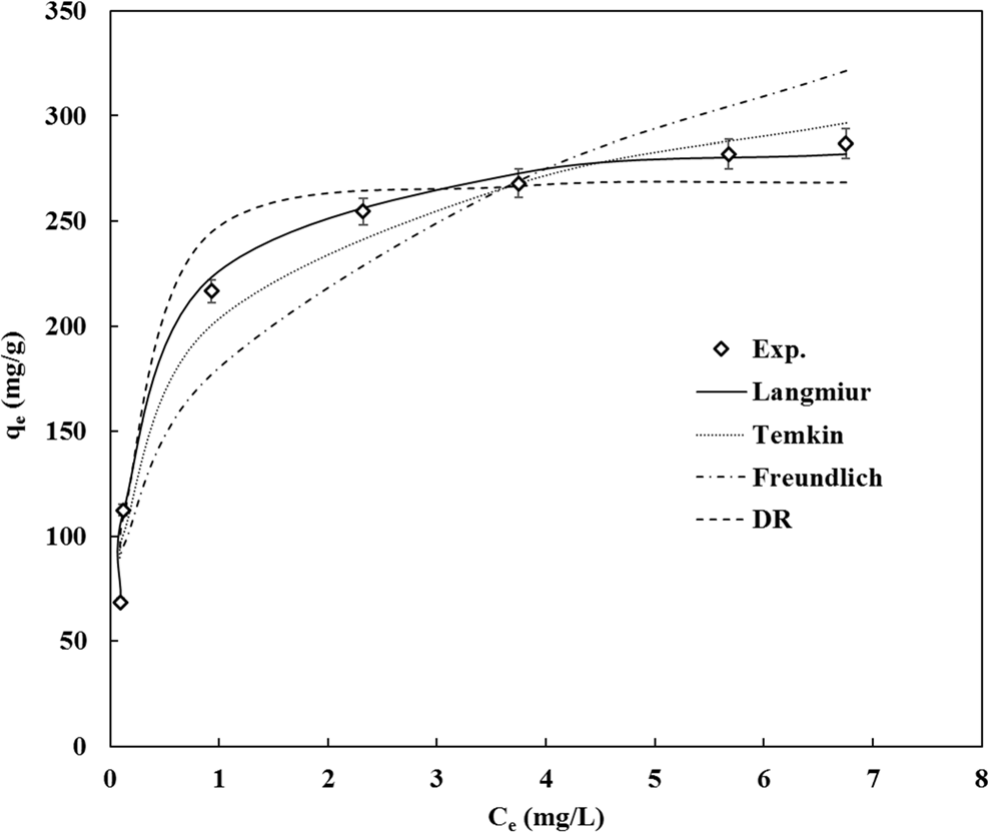
Experimental ibuprofen adsorption data and tested isotherm models; CDC dosage 5 mg, 100 ml of ibuprofen concentrations ranging from 1–21 mg/L at a temperature 20 °C, pH 5, agitation speed 150 RPM and adsorption time 24 h

A range of isotherm types have been utilized for analyzing the equilibrium data; the Langmuir, Temkin, Freundlich and DR isotherm models are commonly used [65, 66]. The adsorption equilibrium and highest adsorption capacity were reached after 24 h of adsorption. The experimental equilibrium data and tested adsorption isotherms of ibuprofen by CDC are presented in Fig. 7. The calculated isotherms parameters and statistical coefficients for all isotherms are given in Tables 4 and 5, respectively. The equilibrium data of ibuprofen adsorption on CDC were well plotted by the Langmuir isotherm model and it was the best in terms of the correlation coefficient (R^2^ > 0.995). To further validate the optimum fit model for correlating the experimental data, the chi-square test (χ^2^) and the sum of the square of the errors (SSE) were evaluated. Smaller values of χ^2^ and SSE indicates better isotherm fitting. Among all tested isotherm models, the Langmuir was in an excellent agreement with ibuprofen equilibrium data at the mentioned conditions, it is not only having the least R^2^ it also has the lowest χ^2^ = 0.44 and SSE = 97.6, these results confirm the adsorption of a monolayer of ibuprofen on the surface of CDC. Moreover, the Langmuir separation factor (R_L_ = 0.015 which is the range 0 < R_L_<1) indicates that this adsorption is favorable [67]. The Freundlich isotherm has the lowest R^2^ value which indicates that the adsorption of ibuprofen on CDC could not be well expressed by this model. Besides, the DR model provides information about the nature of the adsorption by calculating the apparent mean free energy (E). This provides evidence about the adsorption mechanism whether it is physisorption or chemisorption [58]. In our case E = 4.1 kJ/mol, which indicates that the uptake mechanism is dominated by physisorption.

**Table 4:** Results of the isotherms parameters of the adsorption of ibuprofen by CDC, Langmuir, Temkin Freundlich and Dubinin Radushkevich

Langmuir		Temkin	
X_m_ (mg/g) h (L/mg) R_L_	294.1 3.1 0.014	A_t_ (L/mg) b B (J/mol)	72.6 52.5 46.4
Freundlich		Dubinin Radushkevich	
n 1/n K_F_ (mg/g/(mg/L)^1/n^)	3.4 0.3 177.6	q_s_ (mg/g) k_ad_ (mol^2^/J^2^) E (kJ/mol)	262.0 3*10^− 8^ 4.1

**Table 5:** Statistical coefficients of the tested models

Isotherm model	R^2^	χ^2^	SSE
Langmuir	0.999	0.44	97.6
Temkin	0.975	7.71	916.9
Freundlich	0.903	20.35	3481.7
DR	0.961	10.29	1667.8

### Adsorption thermodynamics

Thermodynamic properties (entropy change, enthalpy change and Gibbs free energy change,) of ibuprofen adsorption on CDC were investigated by varying the temperature between 293 and 323 K and the results are illustrated in Table 6. The negative values of the Gibbs free energy point out the spontaneous nature and likelihood of ibuprofen adsorption on the CDC. The change in the Gibbs free energy increases with temperature, which shows that adsorption was enhanced and more favorable at higher temperatures, this could be attributed to the activation of more sites on the CDC surface at higher temperature. The values of the Gibbs free energy were (-12.32 to -16.61 kJ/mol) which fall in the range of -20<$\Delta {G}^{^\circ }$<0 kJ/mol indicating that the adsorption of ibuprofen on CDC is mainly physical, which is in agreement with the result of the apparent free energy value (E) in the DR isotherm model [68]. Otherwise, the adsorption will be chemical if the free energy is in the range − 400< $\Delta {G}^{^\circ }$<-80 kJ/mol. The obtained entropy change was a positive value (142.82 J/mol/K) which represents the disorder and particles freedom enhancement at the CDC water interface throughout the adsorption process. The high value of entropy change may have resulted from the competition between water and ibuprofen molecules on the vacant sites as suggested in some reports [15, 12, 24]. The positive enthalpy change (29.57 kJ/mol) indicates the endothermic nature of the ibuprofen adsorption process.

**Table 6:** Thermodynamic parameters: changes in Gibbs free energy, entropy and enthalpy of ibuprofen adsorption by CDC^*^

Temperature (K)	K_d_	$\Delta {G}^{^\circ }$(kJ/mol)	$\Delta {S}^{^\circ }$ (J/mol/K)	$\Delta {H}^{^\circ }$ (kJ/mol)
293	156.97	-12.32	142.82	29.57
308	280.85	-14.44		
323	483.92	-16.61		

^*^Thermodynamic adsorption tests were examined at pH 5, agitation speed 150 RPM, CDC dosage 5 mg, 100 ml of 5 mg/L ibuprofen

### Adsorption kinetics

**Fig. 8:**
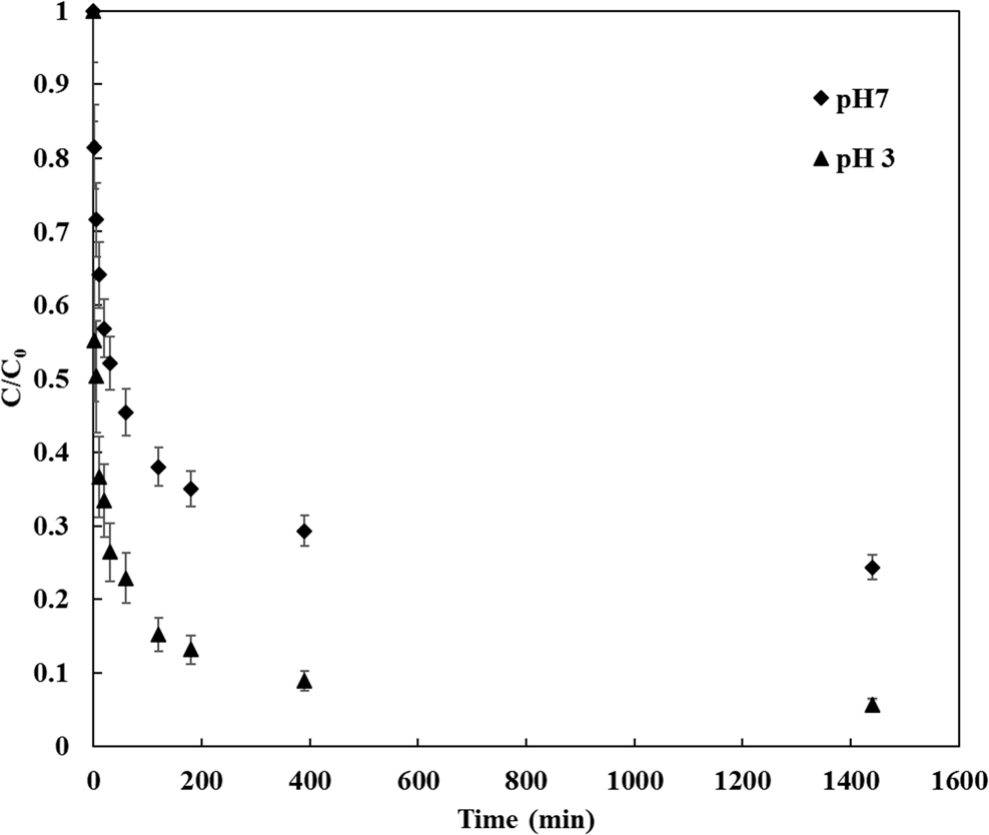
Ibuprofen adsorption kinetic on CDC results, at temperature 20 °C, agitation speed 150 RPM, CDC dosage 5 mg, 100 ml of 15 mg/L ibuprofen

In this study, the ibuprofen adsorption kinetics at CDC were investigated at pH 3 and 7 and the results are illustrated in Fig. 8. It can be seen, that the adsorption was faster in the first 10 min and reached equilibrium in 1440 min contact time for both of the investigated feed pH values. Moreover, it can also be noticed that ibuprofen removal was faster at low pH. For instance, the ibuprofen removal was 63% after 10 min of adsorption at pH 3 while it is 47% at pH 7 indicating that ibuprofen removal is much favorable at low pH. The experimental ibuprofen adsorption data on CDC were correlated with the pseudo 1st and 2nd order kinetic models. Moreover, the analysis of the intra-particle diffusion and Elovich models were also considered. Kinetic constants and the coefficient of determination (R^2^) of each model at pH 3 and pH 7 have been tabulated in Table 7. The determination coefficients of the pseudo 1st order model was 0.89 was lower than the Elovich and the pseudo 2nd order model. Moreover, it was found that the calculated adsorption capacity from the pseudo 1st order model did not match the experimental adsorption capacity of ibuprofen onto CDC. This means that ibuprofen removal by CDC cannot be correlated by the pseudo 1st order kinetics model. The determination coefficient of the intra-particle diffusion model was 0.625 indicating that the adsorption is not controlled by the intra-particle diffusion and the adsorption is dominated by different adsorption mechanisms. The experimental kinetic data were consistent with the pseudo 2nd order kinetic model, which achieved the highest adjusted determination coefficient R^2^ = 0.999 for both pH values (3 and 7). Furthermore, the calculated adsorption value from the pseudo 2nd order model (277.8 mg/g at pH 3 and 222.2 mg/g at pH 7) were closer to the experimental adsorption capacity (276.9 at pH 3 and 222.2 mg/g at pH 7). As illustrated in Table 7, the pseudo 2nd order rate constant (k_2_) was lower for pH 3 meaning that CDC has more affinity to adsorb ibuprofen at lower pH and a larger quantity of CDC was required to adsorb the same amount of ibuprofen at pH 7 as suggested by Guedidi et al. and Mestre et al. [12, 25]. An important factor of the pseudo 2nd order model is the half-life time (t_1/2_) which is the time needed to adsorb 50% of the adsorbate quantity that will be adsorbed at saturation [25]. It offers an explanation regarding the rate of adsorption and can be calculated by the following Eq. (11):

11 $${t}_{1/2}=\frac{1}{{k}_{2}{q}_{e}}$$

**Table 7:** Results of the best fittings of kinetics adsorption models for ibuprofen adsorption by CDC, pseudo 1st order, pseudo 2nd order, Elovich and intra-particle diffusion models

Kinetic model	Factors	pH 3.0	pH 7.0
Pseudo 1^st^ order	q_e_ (mg/g)	108.52	127.06
	k_1_ (min^− 1^)	0.0099	0.088
	R^2^	0.898	0.908
Pseudo 2^nd^ order	q_e_ (mg/g)	277.77	222.22
	k_2_ (g/mg/min)	0.00025	0.00038
	R^2^	0.999	0.999
Elovich	α (mg/g/min)	7091	198.24
	β (g/mg)	0.0395	0.0443
	R^2^	0.955	0.982
Intra-particle diffusion	k_diff_ (mg/g/min^− 0.5^)	3.5244	4.071
	c (mg/g)	177.45	104.22
	R^2^	0.625	0.681

It was found that the half-life period of pH 3 is 9 min while it is 14 min for pH 7, confirming the affinity of ibuprofen adsorption by CDC at the lower pH. Besides the pseudo 2nd order model, the Elovich model can be considered to be in a worthy agreement with the kinetic experimental data, R^2^ = 0.955 and 0.982 for pH 3 and 7, respectively. The Elovich initial adsorption rate (α) was 7091 mg/g/min for pH 3 while it is 198.24 mg/g/min for pH 7. The value (1/ β) representing the number of available sites for adsorption and it is also better for pH values of 3, 1/ β = 25.3 while it is1/ β = 22.5 for pH 7. Moreover; the intercept term of the Elovich model ($\frac{1}{\beta }ln\left(\alpha \beta \right)$) produces the adsorption quantity at ln(t) = zero, i.e. at time = 1 min. This value is important to understand the adsorption process at the initial steps of ibuprofen/CDC contact [69], it is 142.66 mg/g at pH 3 and 49.045 mg/g at pH 7. For an equilibrium adsorption capacity of 277 mg/g at pH 3, it means that more than 50% of the ibuprofen uptake happened after 1 min, which indicates that the removal rate was initially very fast then it decreases with time. This is one of the most attractive attributes of the new material tested in this study, since it implies that more wastewater batches can be treated in a fixed time, and requiring a smaller process plant equipment resulting in a lower capital investment.

### Effect of agitation speed and temperature

**Fig. 9:**
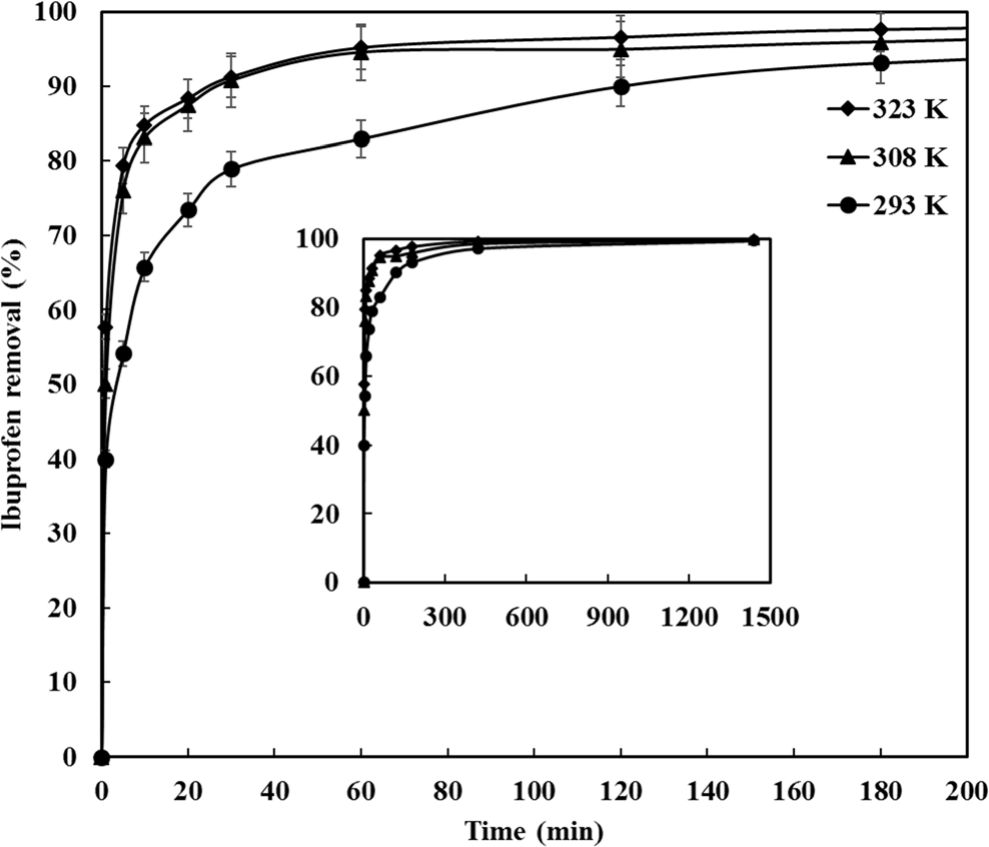
Influence of temperature on the ibuprofen removal from water, ibuprofen concentration 5 mg/L, solution volume 100 ml, CDC loading 5 mg, agitation speed 150 RPM and pH 5

The impact of the temperature on ibuprofen removal by CDC was examined within the temperature range of (293–323 K). As depicted in Fig. 9 the ibuprofen removal improved with the rise in temperature indicating an endothermic adsorption process. The ibuprofen uptake was 40%, 50% and 57% after 1 min at 293, 308 and 323 K, respectively. The difference in ibuprofen removal between 308 and 323 K with time was insignificant, for example; the ibuprofen removal after 10 min of adsorption was 83% and 84% at 308 and 323 K, respectively. The growth in ibuprofen removal with the rise in temperature might be clarified by the fact that increasing temperature will rise the mobility and the rate of mass transfer of ibuprofen molecules to the surface and internal pores of CDC [22]. However, the saturation capacity was similar for all of the investigated temperature. This means that the temperature has affected the rate of ibuprofen removal while having no influence on the adsorption capacity, indicating that the ibuprofen removal by CDC is a physisorption process rather than chemical adsorption.

**Fig. 10:**
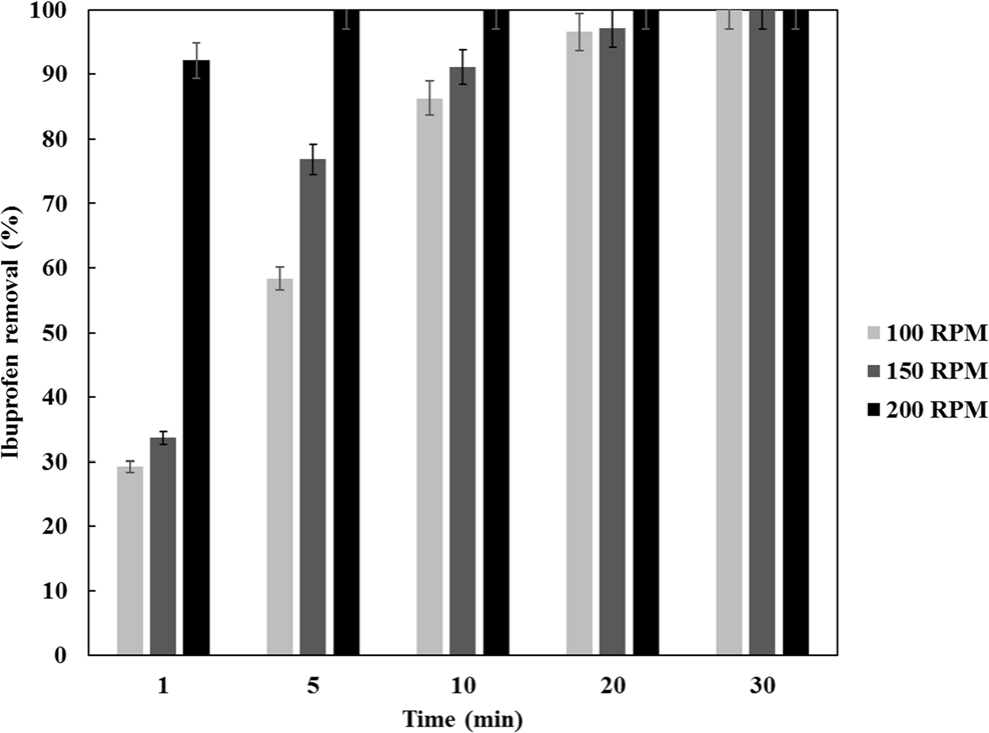
Influence of agitation speed on the removal of removal from water for an ibuprofen concentration 1 mg/L, solution volume 100 ml, CDC loading 5 mg, temperature 20 °C and pH 5

The effect of agitation speed is illustrated in Fig. 10. As shown, the ibuprofen removal improved with agitation speed, this is related to the phenomena that; high agitation speed prevents agglomerations and increases the surface contact between CDC and ibuprofen, hence more collisions happened within the system leading, to better adsorption. The higher the agitation speed this reduces the thickness of the boundary layer surrounding the particle and also enhances the rate of mass transfer of ibuprofen to the particle surface. As depicted from Fig. 10 after 1 min of agitation, the ibuprofen removal was 29.2%, 33.6% and 92.2% at 100, 150 and 200 RPM, respectively. A complete removal of ibuprofen was noticed after 5 min at an agitation speed of 200 RPM, on the other hand; 30 min were required to completely remove the ibuprofen at 100 RPM.

### Effect of initial solution pH and mechanism of adsorption

**Fig. 11:**
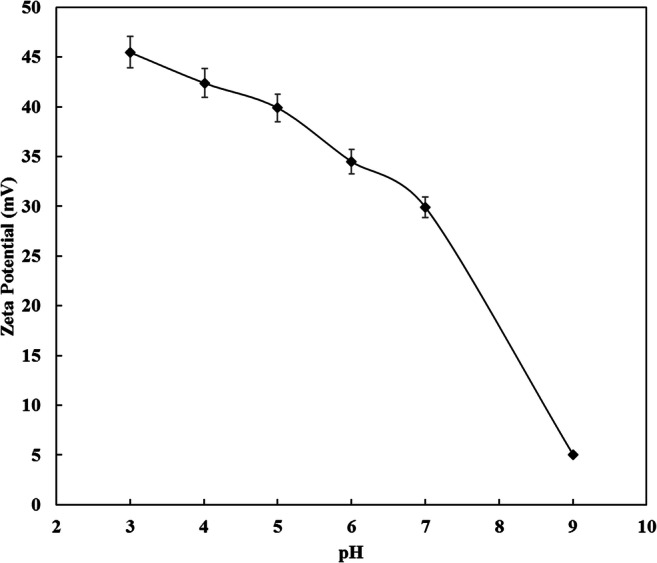
Effect of pH on the zeta potential of CDC

**Fig. 12:**
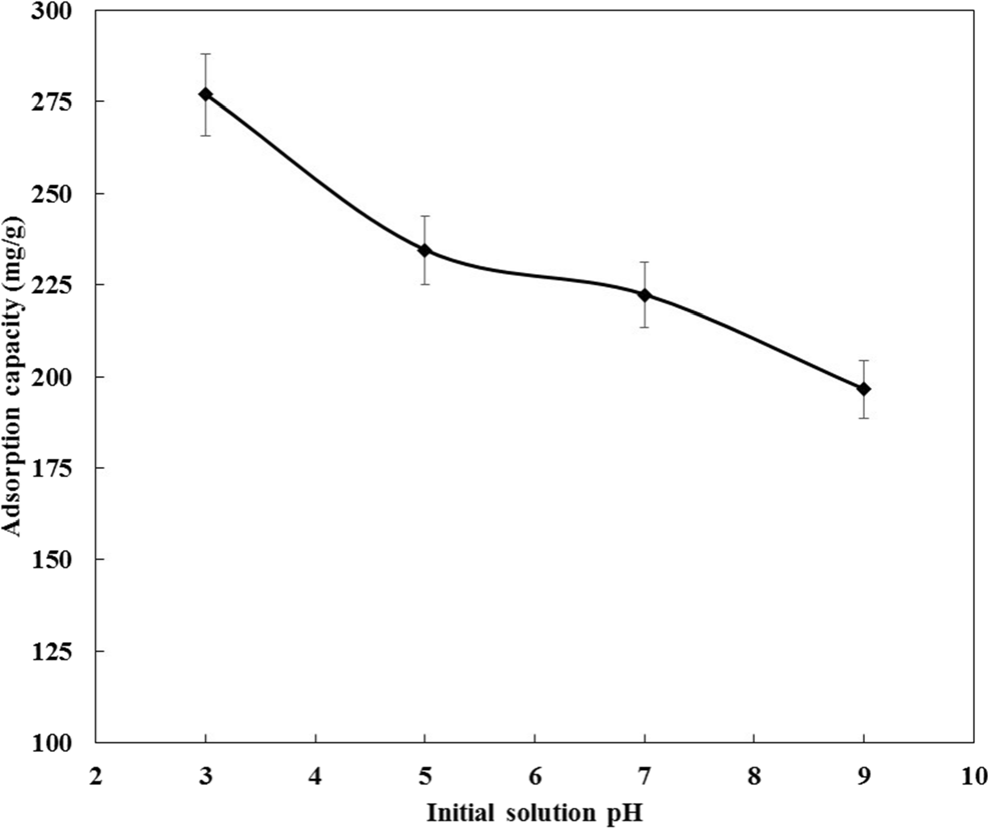
Effectof pH on the adsorption capacity of ibuprofen onto CDC, temperature is 20 °C, agitation speed is 150 RPM, adsorption time is 24 h, CDC dosage is 5 mg in 100 ml of 15 mg/L ibuprofen solution

**Fig. 13:**
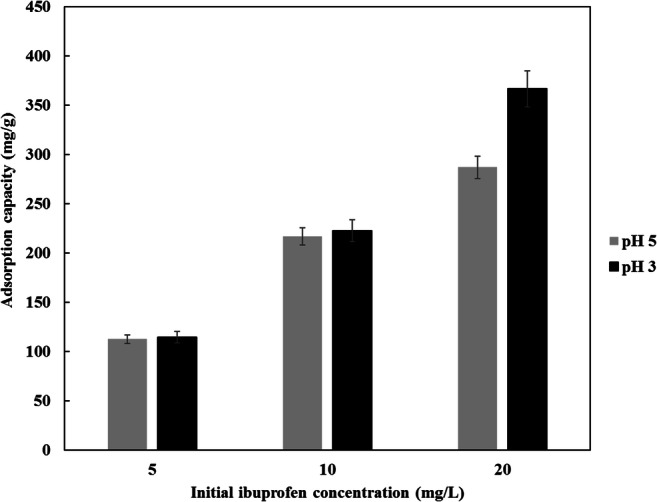
Effect of pH on adsorption capacity at different initial ibuprofen concentration, temperature 20 °C, agitation speed 150 RPM, CDC dosage 5 mg, 100 ml solution and 24 h adsorption

The pH is one of the most important parameters controlling the adsorption capacity of ibuprofen onto carbon based adsorbents. The pH value affects the surface chemistry of the adsorbent and the charge of the adsorbate. Zeta potential measurements of CDC were found to decrease from + 45.5 mV at pH 3 to + 5.0 mV at pH 9 as illustrated in Fig. 11. This means that the pH_PZC_ of the CDC is ˃ 9 which is consistent with the literature [43, 70]. The pKa of the ibuprofen is 4.91 [45]. Figure 12 shows the effect of initial solution pH on the adsorption capacity of ibuprofen onto CDC over the pH range 3 to 9 at an initial concentration of 15 mg/L. It can be seen, the ibuprofen adsorption capacity decreased from 276.9 mg/g at pH 3 to 196.5 mg/g at pH 9. This trend was observed for the ibuprofen removal by the different types of ACs [12, 15, 24, 25]. At pH˃pKa, the ibuprofen molecules are negatively charged and those are adsorbed onto the positively charged CDC by the electrostatic interactions. At pH < pKa, when the CDC particles are still positively charged and ibuprofen species are neutral, the adsorption is less favorable by the electrostatic interactions, and the most dominant adsorption mechanism is by the dispersive interactions [12, 24]. The higher adsorption capacity at low pH could also be attributed to the lower solubility of ibuprofen in acidic media as reported by Shaw et al. [71] that promotes the adsorption of ibuprofen onto CDC. An additional explanation that affects the adsorption of ibuprofen at low pH is the suspension ability of CDC particles in the prepared solution. Higher suspensibility of CDC particles in the solution allows a larger contact area and contact time with the ibuprofen species. At pH 3 the CDC particles charge is more than + 45 mV. This high zeta potential value facilitates good suspensibility of the adsorbent particles, while the CDC surface charge decreases with pH which could accelerate agglomeration of the adsorbent particles at higher values of pH [72, 73]. The maximum ibuprofen adsorption capacity was 367 mg/g at a pH of 3 and 287 mg/g at a pH of 5 using an initial concentration of 21 mg/L of ibuprofen as illustrated in Fig. 13.

### Adsorption of ibuprofen from real TSE

**Fig. 14:**
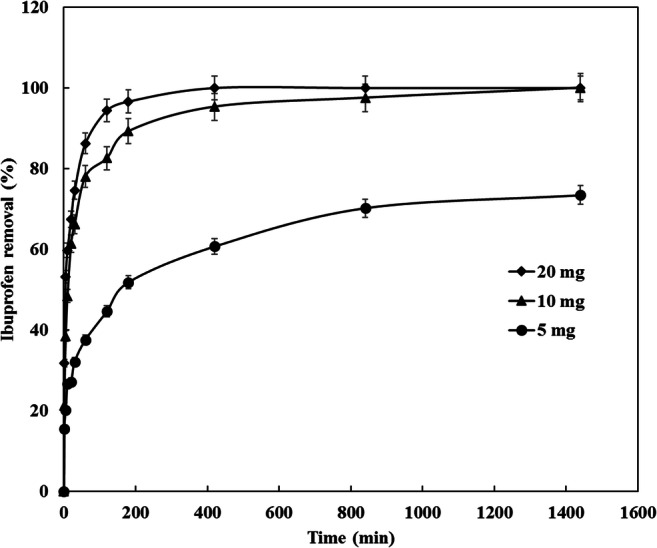
Effect of CDC dosage on ibuprofen removal from TSE, 100 ml, 150 RPM, 20 °C

**Fig. 15:**
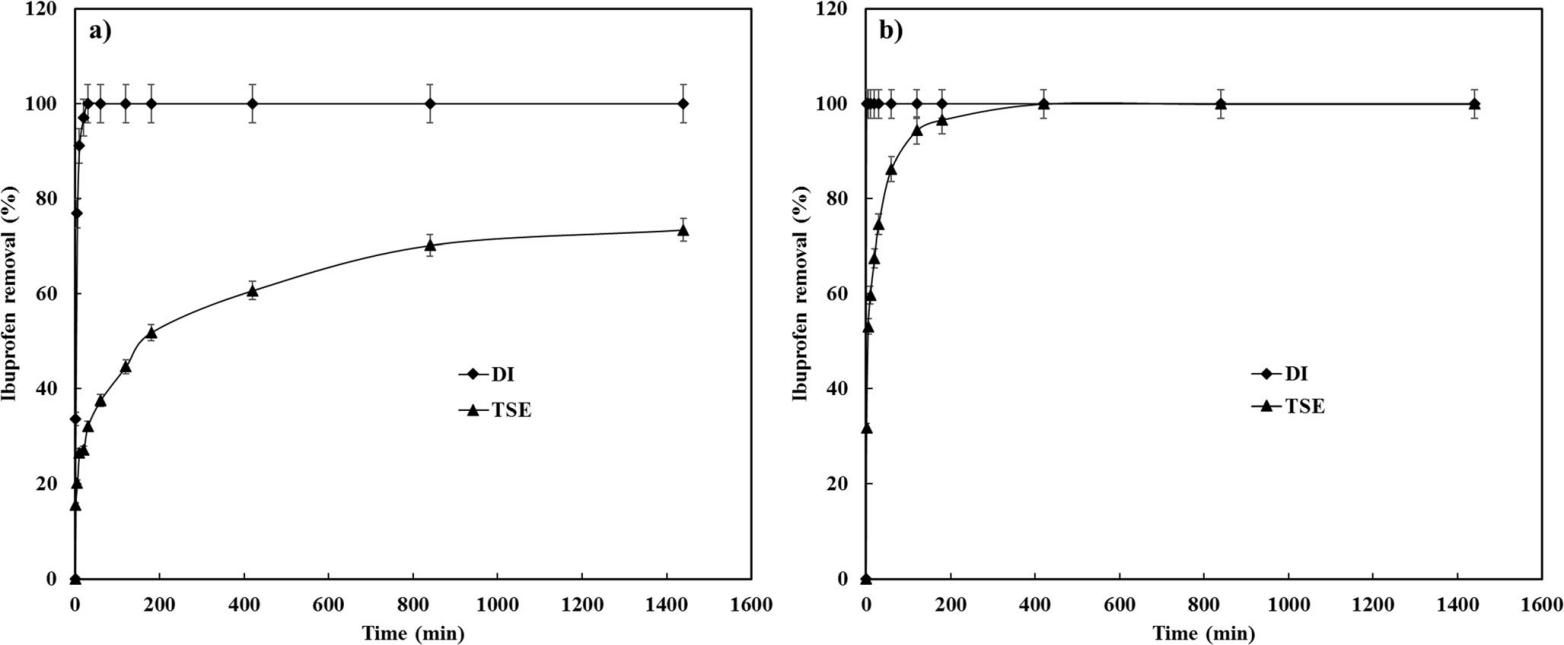
Ibuprofen removal from DI and TSE water at different CDC dosage, (**a**) 5 mg CDC, (**b**) 20 mg CDC, 100 ml, 150 RPM, 20 °C

As stated earlier, the ibuprofen has been found at different concentrations in TSE and surface waters [16]. To investigate the potential real application of CDC, it was used for the treatment of TSE spiked with 1 ppm of ibuprofen. The TSE water properties were as follows; (total organic carbon (TOC) 1.5 ppm, conductivity 2532 MΩ, Phosphate (PO_4_) 4.02 ppm, total phosphorus 1.33 ppm, total dissolved solids (TDS) 1620 ppm). Figure 14 illustrates the ibuprofen uptake from TSE at different CDC dosage. As illustrated, the rate of ibuprofen removal was fast at the beginning of the adsorption process due to the availability of empty sites and plateaued after 800 min. It can also be noticed that the ibuprofen removal increased with CDC loading. For example, the ibuprofen removal was 97% after 3 h using a CDC dosage of 20 mg and a complete removal was achieved after 7 h of adsorption, similarly; a complete removal was achieved using 10 mg of CDC but with a longer adsorption time of about 24 h. A 5 mg dosage of CDC was able to remove 73.4% of the ibuprofen after 24 h of contact time. These results were different from the ibuprofen removal from DI water. Figure 15 depicts the data on ibuprofen removal from TSE and DI at 5 and 20 mg of CDC. The rate of ibuprofen removal from TSE water was lower than the rate of ibuprofen removal from DI water. For instance, using a CDC dosage of 20 mg and the same adsorption conditions, 1 min and 7 h contact time were required to completely remove the ibuprofen from DI and TSE waters, respectively. Furthermore, using 5 mg of CDC required 30 min to completely remove the ibuprofen from DI water, while the maximum uptake was 73.4% from TSE water under similar conditions. Interestingly, using 5 mg of CDC and after 30 min contact time, the CDC was able to remove 32% of ibuprofen from TSE, while it completely removed the ibuprofen from DI water, the CDC has the ability to adsorb 1/3 the amount of ibuprofen from TSE compared to ibuprofen from DI under similar conditions.

The decrease in ibuprofen uptake in TSE compared to DI water might be attributed to several reasons: the blockage of some CDC pores by organics, competition on vacant sites with other organic pollutants, TOC content, the interactions between the ibuprofen and other organic matters in the TSE [74, 75, 43, 76]. However, by using higher quantities of CDC can still lead to complete removal of ibuprofen from TSE.

### Comparison of the CDC adsorption performance with other carbon based adsorbents

Table 8 presents a comparison of the surface area, PZC, isotherm model, equilibrium time, maximum ibuprofen adsorption capacity and the experimental conditions of the reported studies on ibuprofen adsorption removal by carbon based adsorbents such as ACs, CNTs and graphene oxide. It can be noticed that the adsorption capacity of ibuprofen onto the CDC is higher compared to adsorption capacities for most of the used adsorbents except SWCNT and the AC activated K_2_CO_3_ and steam. The differences in the adsorption capacities for these two last adsorbents might come from the used concentrations in the isotherm studies. The concentrations used for the aforementioned adsorbents are in the range up to 2000 and 120 mg/L, respectively, while the highest concentration used in the CDC experiments is 20 mg/L.

**Table 8:** Summary of ibuprofen removal by carbon based adsorbents

Adsorbent	Surface area (m^2^/g)	PZC	Isotherm	Equilibrium time (h)	Investigated ibuprofen concentration (mg/L)	Maximum ibuprofen adsorption capacity (mg/g)	Ref
CDC	1054	˃ 9	Langmuir	24	1–20	367 at pH 3, 293 K	Current study
Commercial granular AC	800	7.76	Langmuir-Freundlich	66.67	5–100	160 at pH 3, 298 K	[12]
H_2_O_2_ oxidized granular AC	781	7.8	Langmuir-Freundlich	-	5–100	146.7, at pH 3, 298 K	[12]
Thermally activated granular AC at 973 K	809	9.08	Langmuir-Freundlich	-	5–100	190.7 at pH 3, 298 K	[12]
MWCNT	283	-	Polanyi Manes model	360	50–2000	186.5 at pH 4, 296 K	[13]
SWCNT	1020	-	Polanyi Manes model	-	50–2000	496.1 at pH 4, 296 K	[13]
Mesoporous Artemisia Vulgaris AC	358.2	5.05	Langmuir	˃ 5	10–50	16.95 at pH 2, 298 K	[15]
Graphene oxide nanoplatelets	-	-	Langmuir	1	2–10	3.72, undefined conditions	[22]
K_2_CO_3_ activated AC	891	7.5	Langmuir	4	20–120	139.2 at pH 4, 298 K	[25]
AC activated by both K_2_CO_3_ and steam	1060	9.9	Langmuir	4	20–120	393.4 at pH 4, 298 K	[25]
Olive waste cake AC	793	5.03	Langmuir		-	10.83 at pH 4.1, 298 K	[77]
Activated charcoal	-	-	Langmuir	2	50–1000	64.5 at pH 4, 298 K	[78]

In general, the adsorption of ibuprofen onto the AC, graphene oxide and CDC follows the Langmuir isotherm model with the exception of granular AC and CNTs. These findings confirm the monolayer coverage of ibuprofen on the carbon based adsorbents. Looking to the adsorption capacities, the highest adsorption capacities were obtained for the adsorbents with the highest surface area and PZC values. For all of the adsorbents, it was noticed that the highest adsorption capacity was obtained at low pH (pH < 4). It should be highlighted that more than of 92% of the ibuprofen saturation capacity onto CDC was achieved after only 3 h of adsorption confirming the competitive kinetics of CDC as a fast adsorbent of ibuprofen.

Considering the fast kinetics and comparable adsorption capacity of CDC, this adsorbent can be considered as a promising material for the removal of pharmaceuticals from aqueous solutions.

## Conclusions

For the first time, CDC has been used as an innovative adsorbent for the removal of ibuprofen from aqueous solutions. The adsorption process parameters were investigated using the batch mode experiments. The surface morphology and the surface area analysis illustrates that produced CDC from a TiC powder precursor is a highly microporous material with an irregular shape and smooth surface. The rate of ibuprofen removal by CDC was enhanced with temperature while the adsorption capacity remained constant indicating a physisorption process. The adsorption results demonstrated that the ibuprofen removal by CDC is highly dependent on the solution pH with a maximum sorption capacity at pH 3. The adsorption process was controlled by the electrostatic interactions at higher pH and by the hydrophobic and dispersive interactions at acidic media. The equilibrium data were following the Langmuir adsorption isotherm while kinetics follows the pseudo 2nd order kinetic model. The change in enthalpy, entropy and Gibbs free energy for the adsorption of ibuprofen by CDC indicates that the process is spontaneous and endothermic in nature. Finally, the CDC was investigated for the removal of ibuprofen from a spiked TSE and it was found that using 20 mg of CDC can achieve a complete removal of 1 mg/L ibuprofen in 100 ml of TSE. These results reveal the capabilities of CDC to be a fast and effective adsorbent of pharmaceutical pollutants removal.

## Data Availability

Not applicable.
